# Faecal microbiota and functional capacity associated with weaning weight in meat rabbits

**DOI:** 10.1111/1751-7915.13485

**Published:** 2019-10-01

**Authors:** Shaoming Fang, Xuan Chen, Liwen Zhou, Chongchong Wang, Qiaohui Chen, Ruiyi Lin, Tianfang Xiao, QianFu Gan

**Affiliations:** ^1^ College of Animal Science Fujian Agriculture and Forestry University Fuzhou China; ^2^ College of Life Science Fujian Agriculture and Forestry University Fuzhou China

## Abstract

Weaning weight is an important economic trait in the meat rabbit industry. Evidence has linked the gut microbiota to health and production performance in rabbits. However, the effect of gut microbiota on meat rabbit weaning weight remains unclear. In this study, we performed 16S rRNA gene sequencing analysis of 135 faecal samples from commercial Ira rabbits. We detected 50 OTUs significantly associated with weaning weight. OTUs that showed positive associations with weaning weight were mostly members of the family *Ruminococcaceae* which are important in degrading dietary fibres and producing butyrate. On the contrary, OTUs annotated to genera *Blautia*, *Lachnoclostridium* and *Butyricicoccus* correlated with fat deposition were negatively associated with weaning weight. Predicted functional capacity analysis revealed that 91 KOs and 26 KEGG pathways exhibited potential correlations with weaning weight. We found that gut microbiota involved in the metabolism of amino acids, butanoate, energy and monosaccharides affected weaning weight. Additionally, cross‐validation analysis indicated that 16.16% of the variation in weaning weight was explained by the gut microbiome. Our findings provide important information to improve weaning weight of meat rabbits by modulating their gut microbiome.

## Introduction

Weaning weight is an important growth trait in commercial meat rabbit breeds. Improving weaning weight could decrease morbidity and mortality in fattening processes and increase economic benefits in the meat rabbit industry. Previous studies have revealed that genetics, diet and health affect weaning weight of meat rabbits (Gomez‐Conde, *et al.*, 2007; Volek, *et al.*, 2007; De Blas, 2013; Drouilhet, *et al.*, 2013; Garreau, *et al.*, 2019). Recently, evidence has suggested that the gut microbiome plays a crucial role in weaning weight modulation (Rosenfeld, 2017; Han, *et al.*, 2017; De Rodas, *et al.*, 2018; Zhou, *et al.*, 2018).

The gut microbiome, which refers to the community of microorganisms that colonizes the intestinal tract, is involved in several physiological processes, such as nutrient metabolism, regulation of the immune system, gut development and protection against pathogens (Combes, *et al.*, 2013; Pickard, *et al.*, 2017). Due to these important functional capacities, several studies have been carried out to understand the vital role of gut microbiota in rabbit health. Dietary fibre is well acknowledged to have many health benefits in rabbits (Gidenne, 2015). Crowley, *et al. *(2017) indicated that both Firmicutes and Bacteroidetes dominate the gut microbial community of rabbits and the shift in ratio of the two phyla may affect the digestive efficiency of dietary fibres. Furthermore, Chen, *et al.* (2019) reported that the abundance of class Alphaproteobacteria may be associated with tolerance against the reduced dietary fibre level in rabbit. On the other hand, Bauerl, *et al. *(2014) suggested that a decrease in the abundance of *Ruminococcus* and *Alistipes* in the gut microbiota of post‐weaning rabbits was related to the development of Epizootic rabbit enteropathy (ERE), one of the most devastating diseases affecting rabbit farms. Conversely, Djukovic, *et al. *(2018) indicated that the disease developed due to an increase in the abundance of *Clostridium cuniculi*.

In addition, the relationships between gut microbiota and production performance of rabbit were uncovered in the previous studies. For instance, Zeng, *et al.* (2015) determined that bacteria, such as YS2, *Bacteroides*, *Lactococcus*, *Lactobacillus* and *Prevotella*, show positive associations with body weight in meat rabbit. Drouilhet, *et al.* (2016) found that propionate‐producing bacteria *Paludibacter propionicigenes* and *Pelotomaculum thermopropionicum* were positively correlated with average daily gain (ADG) and residual feed intake (RFI) in meat rabbit. However, the relationship between gut microbiota and weaning weight in meat rabbits remains unclear.

In the present study, we characterized the profile of faecal microbiota in a commercial Ira rabbit population using high‐throughput 16S rRNA gene sequencing. To identify microbial taxa and potential functional capacities associated with weaning weight, we performed the two‐part model association analysis and Spearman’s correlation analysis, respectively. Moreover, we estimated the contribution of the gut microbiome to the variation of weaning weight. Our results should facilitate the understanding of how gut microbiota affects meat rabbit growth performance and provide information to improve meat rabbit production.

## Results

### Phylogenetic analysis of gut microbiota in meat rabbits

The 16S rRNA gene sequencing generated a total of 20 538 488 reads (range: 94 601–227 632). After quality control and chimera removal, a total of 8 890 596 tags were obtained (range: 41 370–120 796). We clustered the tags into 1460 different operational taxonomic units (OTUs) (range: 539–811).

**Figure 1:**
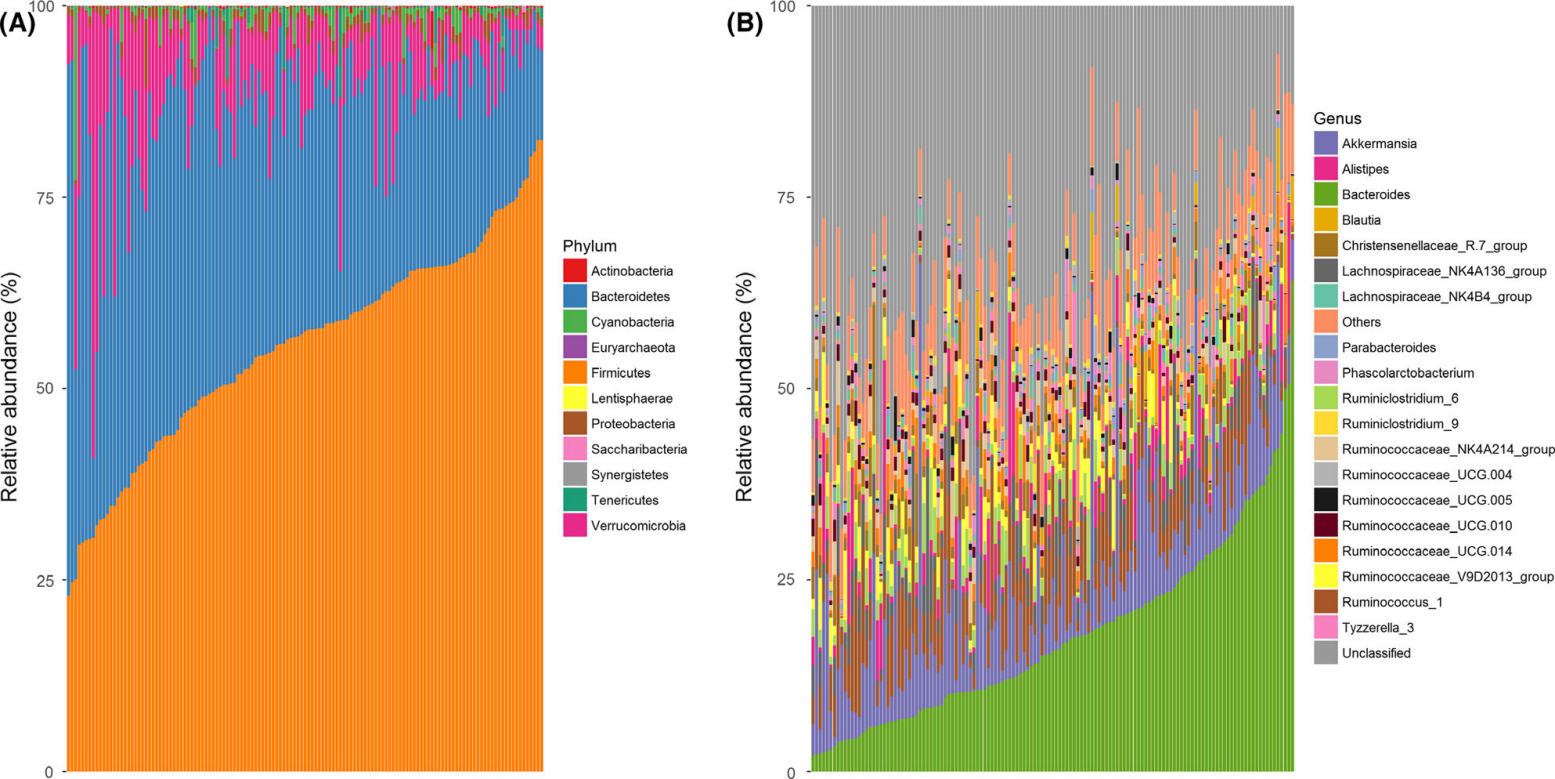
The gut microbial taxonomic distribution in meat rabbit. A. at phylum level. B. at genus level.

Taxonomic assignment indicated the presence of 11 phyla and 78 genera in the meat rabbit gut. Firmicutes, Bacteroidetes and Verrucomicrobia were the three most dominant phyla and accounted for more than 95% of the gut microbial community (Fig. S1). At the genus level, *Bacteroides*, *Akkermansia*, *Ruminococcus_1* and *Lachnospiraceae_NK4A136_group* were the four most predominant genera. However, we also observed that the microbial community composition varied substantially among rabbits. Such as, the average abundance of Firmicutes was 55.39%, ranging from 22.99% to 82.42%. The *Bacteroides* was 17.71%, which varied from 2.08% to 64.11% (Fig. 1).

**Figure 2:**
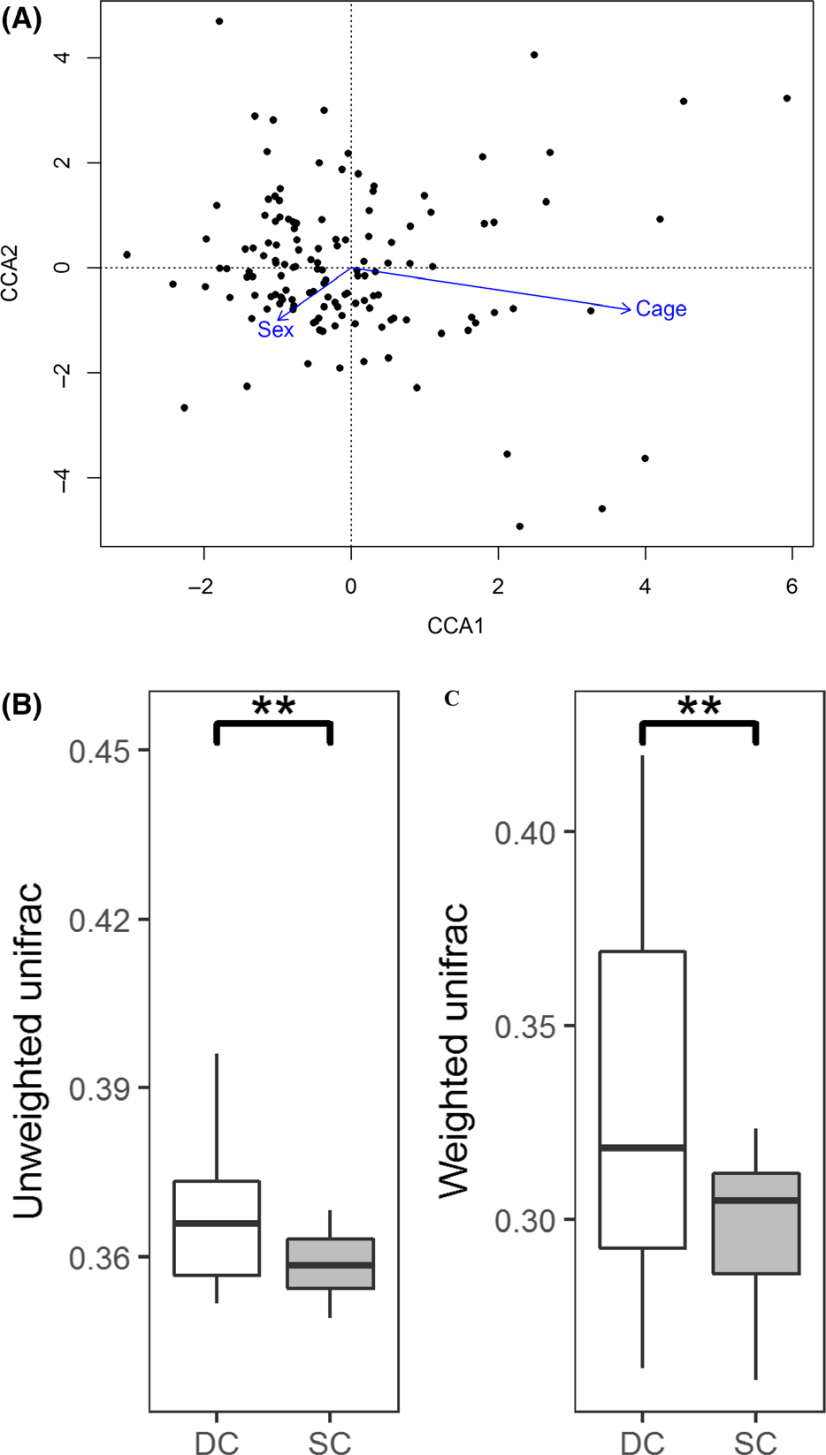
Environmental and host factors affecting meat rabbit gut microbiota composition. A. Canonical Correspondence Analysis (CCA) showed that cage rearing but not sex has significant effect on gut microbial composition. B. The significant effect of cage rearing on Unweighted distance. DC and SC refers to different and same cage respectively. C. The significant effect of cage on weighted UniFrac distance.

Canonical correspondence analysis (CCA) revealed that cage rearing had a significant impact on gut microbial composition of meat rabbits, but sex showed no distinct effect (Fig. 2A). The alpha diversity index analysis also indicated that the microbial community composition did not differ significantly among male and female rabbits (Fig. S2, *P*> 0.05). However, both unweighted and weighted unifrac distance comparison analysis revealed that the microbiotas of rabbits reared in the same cage were more similar to each other than to those of rabbits from different cages (Fig. 2B,C, FDR adjusted *P* < 0.01).

### Microbial taxa associated with weaning weight

To identify microbial taxa associated with weaning weight, we performed a two‐part model analysis using the relative abundances of OTUs and phenotypic values adjusted for sex and cage rearing effects. We identified 50 OTUs associated with weaning weight, of which 19 OTUs showed positive associations and 31 exhibited negative associations (FDR adjusted *P* < 0.05). We annotated these weaning weight associated OTUs to the microbial taxa using SILVA database (Table S1).

**Figure 3:**
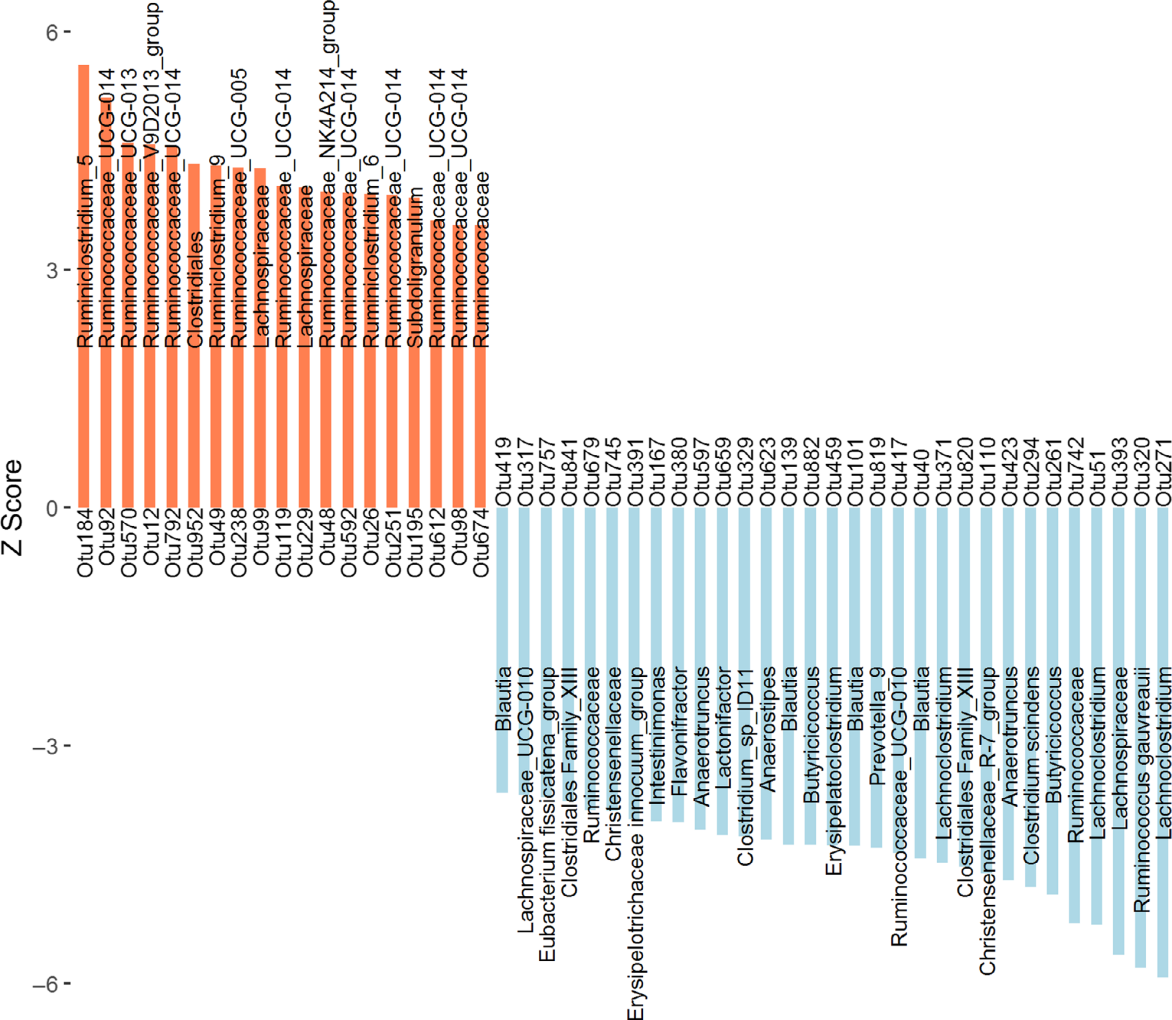
The 50 OTUs showing significant associations with weaning weight (FDR adjusted *P* < 0.05) are shown as *Z *scores. The coral bar represents for positive association, the blue bar corresponds to negative association and the text on the bar shows the microbial taxa annotated to the OUT.

As shown in Fig. 3, among the OTUs positively associated with weaning weight, one OTU was annotated to the order Clostridiales; two OTUs were annotated to the family *Lachnospiraceae* and one to the family *Ruminococcaceae*; seven OTUs were annotated to the genus *Ruminococcaceae_UCG‐014* and one to each of the following genera: *Ruminiclostridium_5*, *Ruminococcaceae_UCG‐013*, *Ruminococcaceae _V9D2013_group*, *Ruminiclostridium_9*, *Ruminococcaceae_UCG‐005*, *Ruminococcaceae_NK4A214_group*, *Ruminiclostridium_6* and *Subdoligranulum*. Among the OTUs negatively associated with weaning weight, six OTUs were annotated to the family level: two to each of *Clostridiales Family_XIII* and *Ruminococcaceae*, and one to each of *Lachnospiraceae* and *Christensenellaceae*. Twenty‐two OTUs were annotated to the genus level: four OTUs to *Blautia*, three OTUs to *Lachnoclostridium*, two to each of *Butyricicoccus* and *Anaerotruncus* and one to each of *Lachnospiraceae_UCG‐010*, *Eubacterium fissicatena_group*, *Erysipelotrichaceae innocuum_group*, *Intestinimonas*, *Flavonifractor*, *Lactonifactor*, *Anaerostipes*, *Erysipelatoclostridium*, *Prevotella_9*, *Ruminococcaceae_UCG‐010* and *Christensenellaceae_R‐7_group*. Three OTUs were annotated to the species level, including *Clostridium_sp_ID11*, *Clostridium scindens* and *Ruminococcus gauvreauii*.

**Figure 4:**
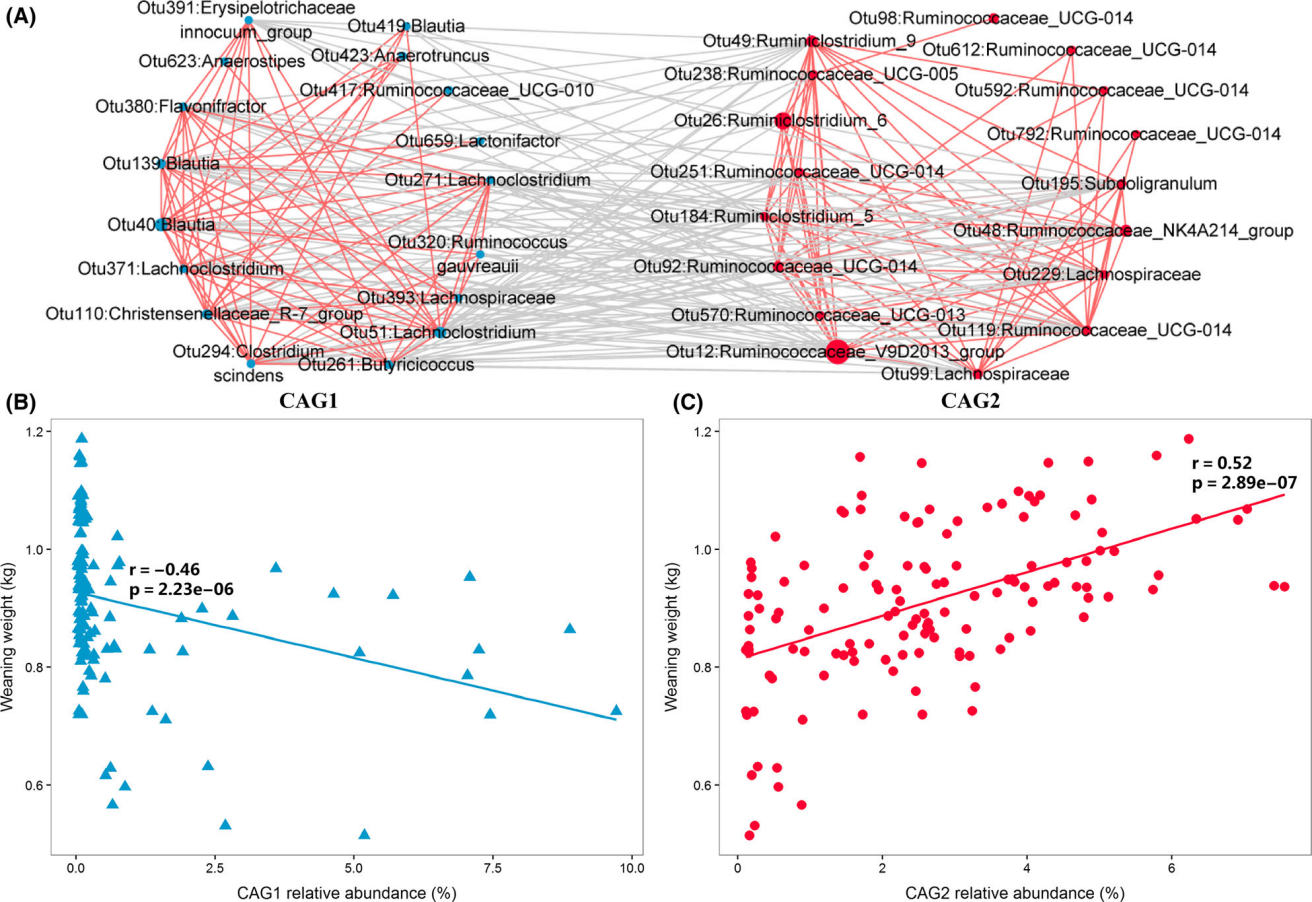
Interactions among the OTUs responding for weaning weight. A. The interaction network of weaning weight associated OTUs. The blue nodes correspond to negatively associated OTUs and the red nodes represent for positively associated OTUs. The node size indicates the average relative abundance of each OTU. Lines linked to nodes indicate significant correlations between the nodes (FDR adjusted *P* < 0.05, |*r*| > 0.4), with red and grey colours showing positive and negative correlation respectively. The OTUs are clustered into two co‐abundance groups (CAGs) by PERMANOVA at *P* < 0.05. B. The correlation between the abundance of CAG 1 and weaning weight. C. The correlation between the abundance of CAG 2 and weaning weight.

To further investigate the potential interactions among the OTUs responding for the weaning weight, the 50 weaning weight associated OTUs were clustered into two co‐abundance groups (CAGs) based on SparCC correlation coefficients (Fig. 4A, Fig. S3). CAG 1 was comprised of the negatively associated OTUs, while the positively associated OTUs formed the CAG 2. These two CAG clusters were negatively correlated with each other. Moreover, we found that CAG 1 was negatively associated with weaning weight, and CAG 2 showed positive association with weaning weight (Fig. 4B,C).

### Predicted functional capacities of gut microbiome related to weaning weight

**Figure 5:**
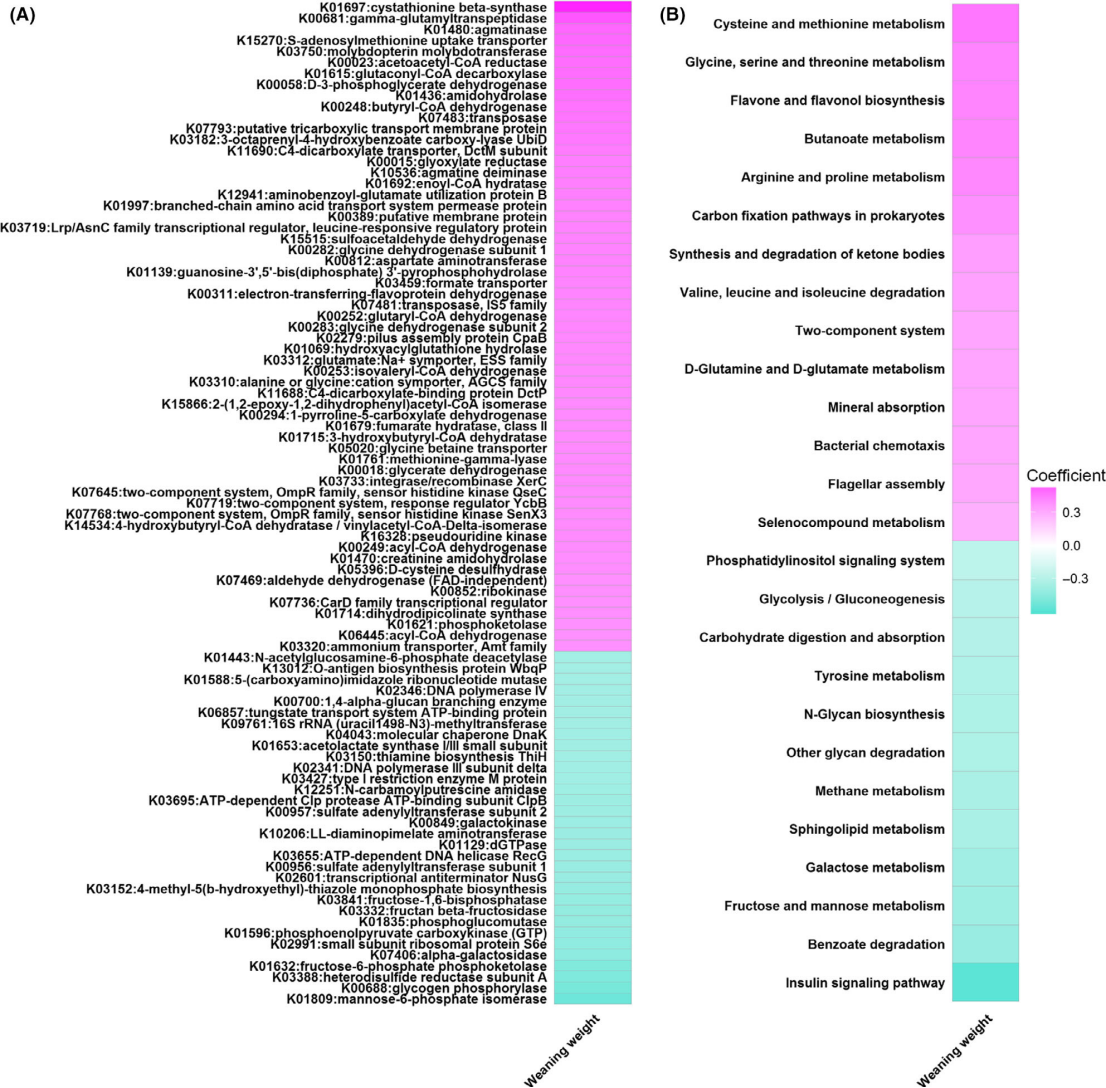
Heatmap of predicted KEGG Orthologs (A) and pathways (B) significantly associated with weaning weight. The correlation coefficient was used for plot.

In order to understand the relationship between the potential functional capacities of gut microbiome and weaning weight, functional profiles of gut microbiome were predicted based on 16S rRNA sequencing data. We identified 91 KEGG Orthologies (KOs) associated with weaning weight with FDR adjusted *P* < 0.05 and |*r*| > 0.3 (Fig. 5A, Table S2). Fifty‐nine out of 91 KOs were positively associated with weaning weight, of which most were related to amino acids metabolism (K01697, K00681, K01480, K00058, K01436, K10536, K01692, K00282, K00812), nutrient transport (K15270, K07793, K11690, K01997, K03459) and butanoate metabolism (K01615, K00023, K00248). The other 32 KOs had negative associations with weaning weight were correlated with energy metabolism (K01809, K01632, K07406, K01835, K03332, K00849) and insulin signalling pathway (K00688, K02991, K01596, K03841).

Additionally, 26 KEGG pathways exhibited significant associations with weaning weight (FDR adjusted *P* < 0.05, |*r*| > 0.3, Fig. 5B, Table S3). Fourteen pathways had positive associations with weaning weight, including cysteine and methionine metabolism, glycine, serine and threonine metabolism, flavone and flavonol biosynthesis, butanoate metabolism and arginine and proline metabolism. Meanwhile, 12 pathways were negatively associated with weaning weight, such as, insulin signalling pathway, benzoate degradation, fructose and mannose metabolism and galactose metabolism.

### Phenotypic variation of weaning weight explained by gut microbiome

**Figure 6:**
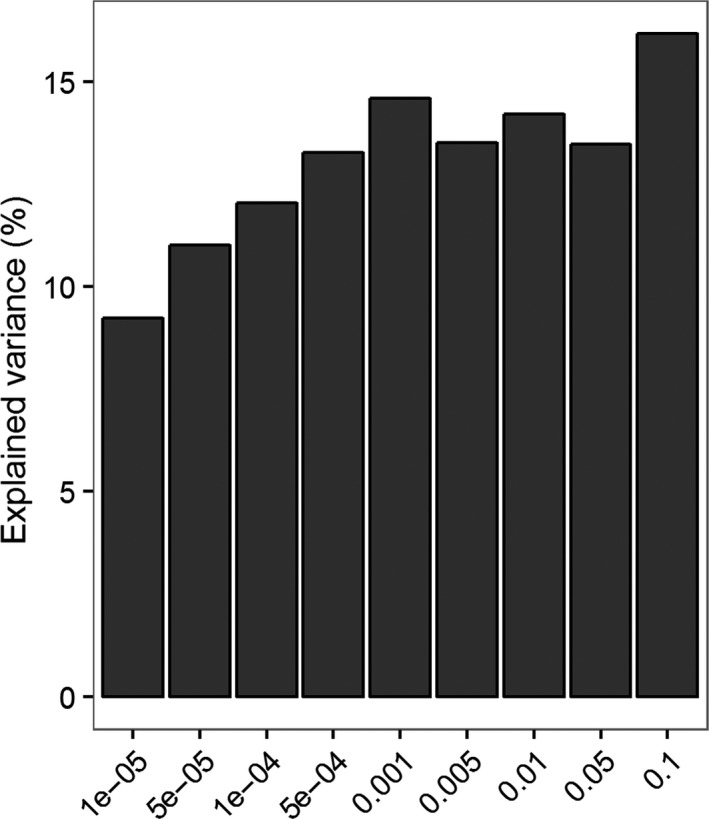
The variation of weaning weight explained by gut microbiome at different *P* values.

To investigate the contribution of gut microbiome to the variation of weaning weight, we performed 100 times cross‐validation analyses at different *P* value thresholds (ranging from 10^−5^ to 0.1). As shown in Fig. 6, we found that the OTUs identified at *P* = 1.0 × 10^−5^ could explain 9.22 % of the variations in weaning weight. With *P* = 0.1, the gut microbiome explained 16.16% of the variations in weaning weight given that as the threshold increased, more OTUs were included in the analysis.

## Discussion

Recently, studies have indicated that gut microbiota plays a crucial role in the host’s nutrient metabolism, immune system modulation and development of diseases. Hence, gaining insight into the relationship between gut microbiota and the livestock physiology is essential to improve their production performances. However, the role of gut microbiota in meat rabbit weaning weight is still elusive. To our best knowledge, this is the first comprehensive study on the relationship between gut microbial community and meat rabbit weaning weight in terms of the microbial taxa and the potential functional capacities through 16S rRNA gene sequencing.

In line with previous studies on the gut microbiota of rabbits (Velasco‐Galilea, *et al.*, 2018; Chen, *et al.*, 2019), we found that Firmicutes and *Bacteroides* were the most abundant phylum and genus in the gut microbiota of meat rabbit, respectively (Fig. S1). However, the relative abundance of each phylum and genus differed substantially among samples (Fig. 1), suggesting a high variability in the gut microbial phylogenies between individuals. Due to the fact that weaning rabbits are sexually immature, we found that cage rearing but not sex could significantly affect the gut microbial composition (Fig. 2, Fig. S2). Earlier studies indicated that the cage rearing effect may be caused by differences in early life gut microbial colonization process, including exposure to the maternal microbiome (e.g. breast‐milk feeding and ingest the soft faeces of dams) and indigenous environment microbiome (Hufeldt, *et al.*, 2010; Rogers, *et al.*, 2014; Ericsson, *et al.*, 2015; Yang, *et al.*, 2017; Korpela, *et al.*, 2018; Martinez, *et al.*, 2018). Previous studies investigated the role of sex in adult animals’ gut microbiota suggested that the significant difference of gut microbial composition between sexes was caused by sex hormones (Org, *et al.*, 2016; Miyoshi, *et al.*, 2018). However, Steegenga, *et al.* (2014) indicated that these sexual microbial differences do not present in the young animals and are only detected after puberty. This could explain why no significant effect of sex on meat rabbit gut microbial composition was observed in our study.

In total, we identified 50 OTUs that had significant associations with weaning weight (Fig. 3, Table S1). Most of the weaning weight positively associated OTUs were annotated to the members of the family *Ruminococcaceae*, such as *Ruminococcaceae_UCG‐014*, *Ruminiclostridium_5* and *Ruminococcaceae_UCG‐005* which have been reported to produce butyrate during fermentation of dietary indigestible fibre and polysaccharides (Louis, *et al.*, 2010; Chen, *et al.*, 2017; Zhao, *et al.*, 2017). Butyrate generated by gut microbiota represent a major energy source for both host colonic epithelium and gut microbes that contributes to the maintenance of the gut barrier functions (Bui, *et al.*, 2015; Riviere, *et al.*, 2016). Importantly, butyrate acts as a signal transduction molecule of G protein‐coupled receptors (FFAR3, GPR109A), involved not only in regulating energy and nutrient metabolism, but also having an anti‐inflammatory effect (Le Poul, *et al.*, 2003; Ahmed, *et al.*, 2009; Kasubuchi, *et al.*, 2015). On the contrary, we found that OTUs annotated to genera *Blautia*, *Lachnoclostridium*, *Butyricicoccus*, *Anaerotruncus* and *Ruminococcus gauvreauii* were negatively associated with weaning weight. These bacteria have been related to chronic low‐grade inflammation and metabolic diseases (e.g. obesity and type 2 diabetes) in humans and rats (Pataky, *et al.*, 2016; Guo, *et al.*, 2018; Tang, *et al.*, 2018; Kong, *et al.*, 2019). In rabbits, low‐grade inflammation and fat deposition have been known to negatively impact growth (Sigrist‐Flores, *et al.*, 2019). Interestingly, we did not observe bacteria associated with the metabolism of breast milk, such as *Bifidobacterium*, *Lactobacillus*, *Enterococcus* and *Staphylococcus* exhibit significant associations with weaning weight (Jost, *et al.*, 2015; de Muinck and Trosvik, 2018). This could be due to young rabbits feeding off their mothers’ solid feed at an early age (15‐day‐old pups). The abundances of bacteria that degrade solid feed (e.g. *Ruminococcaceae*) increase with age and become more dominant as the weaning period comes to an end (Padilha, *et al.*, 1995). Hence, we believe that gut microbiota containing *Ruminococcaceae* species should play a vital role in meat rabbit weaning weight.

Co‐abundance groups analysis revealed that co‐occurrence and competitive relationships exist among microbial taxa associated with weaning weight and that such competitive interactions could affect weaning weight (Fig. 4). Similarly, other studies have reported that gut microbial interactions are essential in modulating body weight of meat producing animals. For instance, Ramayo‐Caldas, *et al. *(2016) suggested that a strong co‐exclusion between *Prevotella* and *Ruminococcus* in the interaction network of gut microbiota significantly affect porcine body weight. In another pig gut microbiota study, a strong co‐exclusion between *Enterobacteriaceae* and *Prevotellaceae* related to the metabolism of SCFAs and energy provides an important direction for manipulating gut microbiota to improve body weight (Zhang, *et al.*, 2018). Additionally, both Broom *et al.* and Ma *et al.* emphasized the importance of gut microbial interactions in production performances improvement of broiler chickens (Broom and Kogut, 2018; Ma, *et al.*, 2018).

We identified 91 KOs and 26 KEGG pathways significantly associated with weaning weight. Among these, those mostly related to the metabolism of amino acids and butanoate have positive associations with weaning weight (Fig. 5, Table S2 and S3). The important effects of gut microbial amino acid and butanoate metabolism on animals’ production performance have been demonstrated in earlier studies. Jiao *et al.* indicated that cysteine and methionine metabolism mediated by gut microbiota significantly affects average daily gain of young cattle (Jiao, *et al.*, 2017). Guevarra, *et al.* (2018) reported that gut microbiota involved in arginine and proline metabolism could alleviate weaning stress and improve growth performance in piglet. Li, *et al. *(2018) suggested that the metabolism of butanoate modulated by gut microbiota had influence on porcine weaning weight. On the contrary, KOs correlated with energy metabolism and KEGG pathways related to monosaccharide metabolism showed negative correlations with weaning weight. Many studies have suggested that gut microbiome with more capacity to harvest energy would increase body fat accumulation, which could then hinder growth hormone secretion resulting in reduced growth performance (Scacchi, *et al.*, 1999; Scheithauer, *et al.*, 2016; Hall and Versalovic, 2018; Velasquez, 2018; Dimitri, 2019; Li, *et al.*, 2019). Moreover, some monosaccharides (e.g. glucose, fructose, mannose and galactose) could inhibit the expression of genes encoding cellulases and xylanases in gut bacteria, which would decrease the degradation of polysaccharides and the production of short chain fatty acids (SCFAs) (Nguyen, *et al.*, 1997; Rodriguez, *et al.*, 2005; Han, *et al.*, 2017). The insufficient amounts of SCFAs have negative impact on inducing the synthesis of insulin like growth factor 1 (IGF‐1) which may affect growth performance via IGF‐1/GH axis (Du, *et al.*, 2018; Schwarzer, *et al.*, 2018; Yan and Charles, 2018).

As shown in Fig. 6, we estimated that gut microbiome could explain 9.22–16.16% of the variation in weaning weight, and these effects are similar to the effects that host genetics have on weaning weight (8.3–13.9%) (Krogmeier, *et al.*, 1994; Lukefahr, *et al.*, 1996). This suggests that we should pay more attention to the effect of gut microbiome in the rabbits for both breeding and meat production purposes.

We performed a 16S rRNA gene sequencing to investigate diversity and composition of gut microbial community, but it has great limitations in resolution and accuracy at the species and strains levels. Although we predicted functional profiles using 16S rRNA gene sequencing data, it only provides some reference information for gut microbial genes and functions. Hence, based on our results, firstly, the metagenomic sequencing analysis should be performed to identify species and functional capacities that affect weaning weight. Then, high‐throughput microorganism culture system would be applied to isolate species and strains that exert significant effects on weaning weight. Finally, the isolated bacteria could be used for specific‐pathogen‐free (SPF) rabbit intervention trial to examine causal relationship between specific species and weaning weight. These works are combined with other omics’ investigations (e.g. metabolome and transcriptome) will illustrate the mechanisms of how gut microbiome affects weaning weight and provides important potential probiotic sources for improving weaning weight in the meat rabbit production.

In conclusion, we detected 50 OTUs significantly associated with weaning weight in meat rabbits. We found that members of the family *Ruminococcaceae*, which are involved in degrading dietary indigestible fibres and polysaccharides and in producing butyrate, were important in improving weaning weight. Predicted functional capacities analysis further uncovered the potential effects of gut microbiota involved in the metabolism of amino acids, butanoate, energy and monosaccharides on weaning weight. Cross‐validation analysis indicated that gut microbiome had a similar effect size on weaning weight as host genetics. Thus, our results provide an important insight into how gut microbiota influences weaning weight. Additionally, we have revealed some microorganisms that could be potentially used to improve weaning weight in meat rabbit industry.

## Experimental procedures

### Experimental animals and sample collection

A total of 135 weaning Ira rabbits (70 males and 65 females) were used in this study. Six to eight pup rabbits were raised with their mothers per cage under natural light and room temperature in the same commercial farm. A commercial pellet diet (details are shown in Table S4) was provided to lactation does two times a day, and pup rabbits had free access to the feed. Pup rabbits were weaned with 28 ± 2 days, at which point one or two rabbits were randomly selected from 90 cages for body weight measuring. The weaning rabbits were healthy and had not received antibiotics, anticoccidial drugs, probiotics or prebiotics before sampling. All hard faecal samples were collected from the anus of weaning rabbits using sterilized EP tubes and immediately dipped in liquid nitrogen for transportation. In the laboratory, faecal samples were stored at −80°C until use.

### Microbial DNA extraction and 16S rRNA gene sequencing

Microbial DNA was extracted from faeces using the QIAamp Fast DNA Stool Mini Kit (Qiagen, Hilden, Germany) following the manufacturer’s protocol. For each sample, the purity and integrity of total DNA were assessed using the Nanodrop ND‐2000 spectrophotometer (Thermo Fisher Scientific, Waltham, MA, USA) and 1.5% agarose gel electrophoresis respectively. The qualified DNA samples were used for downstream PCR and sequencing steps.

The V3‐V4 hypervariable region of the 16S rRNA gene was amplified by the barcoded fusion primers 341F (5’‐CCTACGGGNGGCWGCAG‐3’) and 806R (5’‐GGACTACHVGGGTATCTAAT‐3’). PCR amplification procedure consisted of an initial denaturation step at 95°C for 3 min, followed by 28 cycles of 95°C for 30 s, 55°C for 30 s and 72°C for 30 s, and a final extension step at 72°C for 10 min. The Agencourt AMPure XP system (Beckman Coulter, Brea, CA, USA) was used to purify the PCR products. The final DNA libraries were sequenced on a Hiseq‐2500 platform (Illumina, San Diego, CA, USA) according to the manufacturer’s manuals.

### 16S rRNA gene sequencing data analysis

To obtain the clean data, the primers, barcodes and low quality sequences were removed from the raw sequencing data. High‐quality paired‐end reads were merged to produce tags using flash (v.1.2.11) (Magoc and Salzberg, 2011). To normalize the sequencing depth, we rarefied the library size of microbial sequences to 40 000 tags per sample before further analysis. usearch (v.10.0) was used for picking OTUs at 97% sequence identity (Edgar, 2010). We filtered out those OTUs which had relative abundance < 0.05% and were presented in < 5% of the experimental rabbits from further analysis. We performed the taxonomic assignments of OTUs against SILVA database (v.132) using the Naïve Bayesian algorithm (Wang, *et al.*, 2007; Quast, *et al.*, 2013). The alpha and beta diversity indices were calculated using mothur (v.1.41.1) and qiime (v.1.9.1) respectively (Schloss, *et al.*, 2009; Caporaso, *et al.*, 2010).

### Statistical analysis

In order to identify the effect of cage rearing and sex on gut microbial communities, CCA was performed using the vegan package in r software (He, *et al.*, 2016). The Shannon and observed species index of alpha diversity were compared between males and females using the Wilcoxon test with false discovery rate (FDR) correction. The Wilcoxon test with FDR correction was also used to detect differences in both unweighted and weighted unifrac distance comparisons between rabbits raised in the same cage and those raised in the different cages.

Residuals of weaning weight phenotypic values corrected the effects of sex and cage rearing and were used for further association analysis between weaning weight and relative abundances of OTUs. Because the relative abundances of OTUs showed a non‐normal distribution, the association analysis was performed using a two‐part model method as described previously (Fu, *et al.*, 2015). Briefly, the two‐part model association analysis consisted of a binary model and a quantitative model. The binary model performs a binomial analysis that assesses for the effect of the presence/absence of the gut microbe on weaning weight. The quantitative model analysed the association between weaning weight and the relative abundance of OTUs, but only the samples where that microbe was detected were included in the analysis. A meta‐analysis was also performed using an unweighted Z method to test for the effect of both binary and quantitative features. The final association *P* value was set as the minimum of *P* values derived from binary, quantitative and meta‐analysis. In order to correct the FDR of multiple tests, 1000 × permutations were performed and FDR adjusted *P* < 0.05 was set as the significance threshold. The Sparse Correlations for Compositional data (SparCC) algorithm was used for analysing correlation among weaning weight associated OTUs (Friedman and Alm, 2012). These correlations were controlled for multiple testing using a bootstrap produce, and only those with *P* < 0.05 and |*r*| > 0.4 were retained and visualized using cytoscape (v.3.7.0). To cluster the weaning weight associated OTUs into co‐abundance groups (CAGs), the Ward clustering method and PERMANOVA (999 permutations, *P* < 0.05) based on SparCC correlation coefficients were perfomed by using the made4 and vegan r package respectively (Odamaki, *et al.*, 2016; Zhang, *et al.*, 2016). Spearman’s correlation analysis with FDR correction was performed to identify the association between the abundance of CAGs and weaning weight phenotypic values (Liu, *et al.*, 2017).

Potential functional capacities of gut microbial community were predicted with the tax4fun software using the 16S rRNA gene sequencing data as described by Asshauer *et al.* (Asshauer, *et al.*, 2015). In brief, the SILVA‐based 16S rRNA profile is transformed to a taxonomic profile of prokaryotic KEGG organisms using the linear transformation method. Then, 16S rRNA copy number derived from the NCBI gene annotations was used for normalizing the estimated abundances of KEGG organisms. Finally, the potential functional profile of microbial community was predicted by linearly combining the normalized taxonomic abundances to the precomputed functional profiles of the KEGG organisms. The association between the relative abundances of potential function capacities (KEGG pathways and KEGG Orthologies) and weaning weight phenotypic values was analysed using the Spearman’s method with FDR correction.

To investigate the contribution of gut microbiome to the variation of weaning weight, a 100 × cross‐validation was performed as described by Fu *et al. *(2015). We randomly divided the data set into an 80% discovery dataset and a 20% validation data set. In the discovery data set, the two‐part model association analysis were performed to identify a number of (*n*) OTUs that were significantly associated with phenotype at a certain *P* value and assessed the effect sizes of binary and quantitative features (β_1_ and β_2_) of each OTUs. In the validation data set, the effect of gut microbiome on weaning weight (*r*_m_) for each individual was estimated by an additive model: *r*_m_ = $\sum_{j=1}^{n}\left(\beta_{1}+b_{j}+\beta_{2}q_{j}\right)$, where *b_j_* and *q_j_* represent the binary and quantitative feature of *j* OTU respectively. We calculated the squared correlation coefficient (*R* ^2^) between *r*_m_ and the phenotypic value (corrected for sex and cage), which represents the phenotypic variance explained by the gut microbiome. We repeated the cross‐validation for 100 times and calculated the average value of the explained variations to ensure validity and stability of the estimation.

## Conflict of interest

None declared.

## Availability of sequencing data

We submitted 16S rRNA gene sequencing data to the SRA database in NCBI with accession numbers: SRR8898360, SRR8898359, SRR8898362, SRR8898361, SRR8898364 and SRR8898363.

## Authors’ contributions

TX: conceived and designed the experiments, supervised the experiment progress and revised the manuscript; QG: designed the experiments, analysed the data, wrote and revised the manuscript; SF and XC: performed the experiments, analysed the data and wrote the manuscript; LZ, CW and QC: performed the experiments; RL: revised the manuscript. All authors read and approved the final manuscript.

## Ethical approval

All animal works were conducted according to the guidelines for the care and use of experimental animals established by the Ministry of Agriculture and Rural Affairs of China. The project was specially approved by Animal Care and Use Committee (ACUC) in Fujian Agriculture and Forestry University.

## Supporting information


**Fig. S1.** The relative abundance of phylum and genus in gut microbial community of meat rabbit.

**Fig. S2.** The comparison of observed species and Shannon index between males and females.

**Fig. S3.** The weaning weight associated OTUs formed two clusters using Ward clustering algorithm.

**Table S1.** OTUs showing significant associations with weaning weight and taxonomic assignment using SILVA database.

**Table S2.** KEGG Orthologies (KOs) showing significant associations with weaning weight.

**Table S3.** KEGG pathways showing significant associations with weaning weight.

**Table S4.** Composition of pellet diet of lactating does.
