# Supplementary figures and images for: Faecal microbiota and functional capacity associated with weaning weight in meat rabbits
Source: Microb Biotechnol. 2019 Oct 1;12(6):1441–52. doi: 10.1111/1751-7915.13485 (PMC6801154; doi:10.1111/1751-7915.13485)

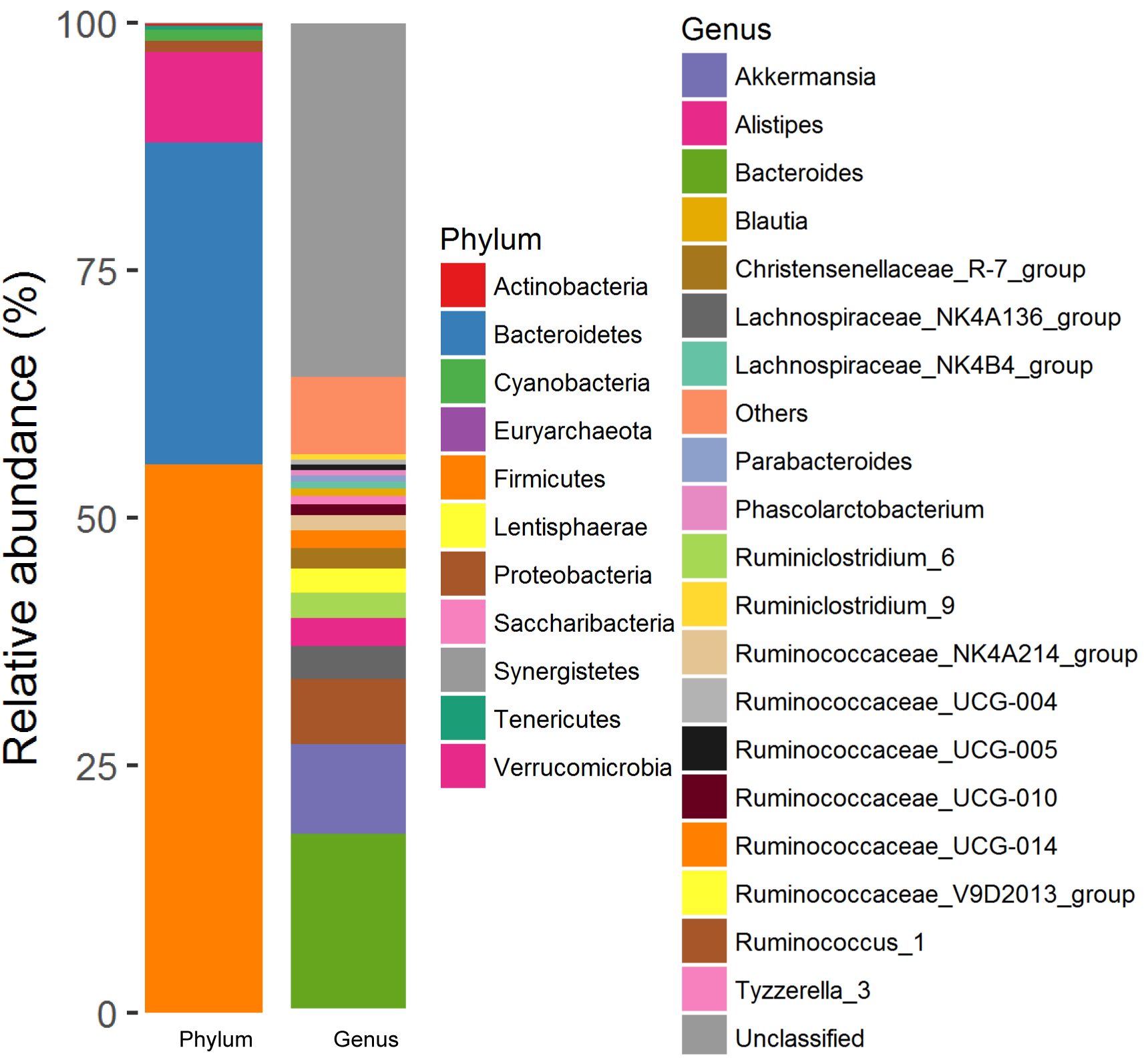

Supplement: Supplementary file 1 — Fig. S1. The relative abundance of phylum and genus in gut microbial community of meat rabbit. [file MBT2-12-1441-s001.tiff]

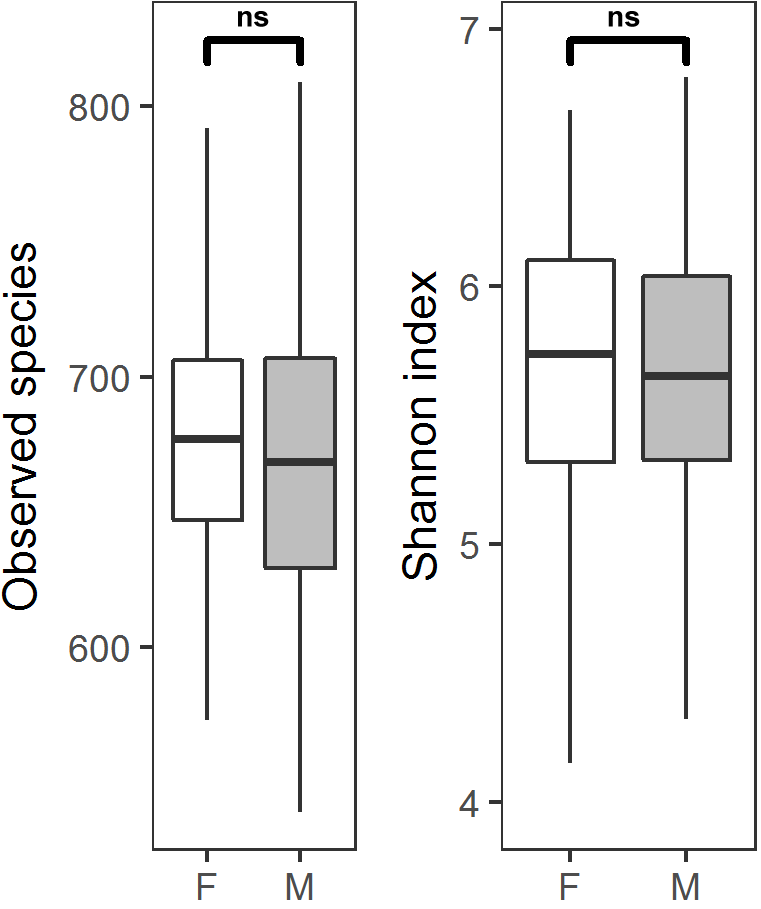

Supplement: Supplementary file 2 — Fig. S2. The comparison of observed species and Shannon index between males and females. [file MBT2-12-1441-s002.tiff]

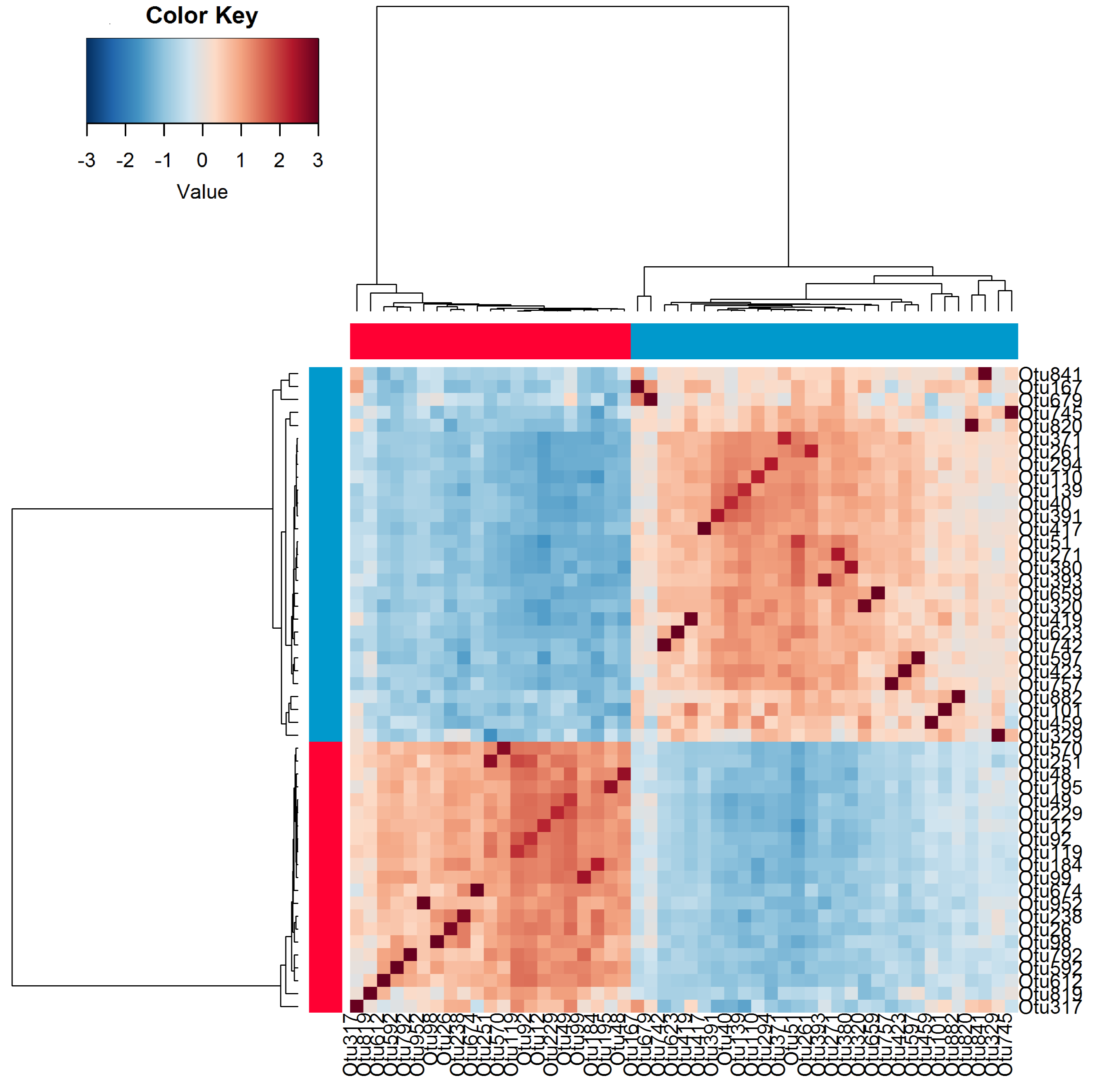

Supplement: Supplementary file 3 — Fig. S3. The weaning weight associated OTUs formed two clusters using Ward clustering algorithm. [file MBT2-12-1441-s003.tiff]
